# Health-Related Fitness of Adolescent Boys with Type 1 Diabetes Mellitus After Recreational Football Exercise with Caloric Control

**DOI:** 10.1900/RDS.2023.19.77

**Published:** 2023-06-30

**Authors:** Mohammed Hamdan Hashem Mohammed, Mohammad Hussain Hassan Al-Qahtani, Tim Takken

**Affiliations:** 1 PhD, Physical Education Department, King Fahd University of Petroleum & Minerals, Dhahran 31261, Saudi Arabia,; 2 MD, Department of Pediatrics, Imam Abdulrahman Bin Faisal University, Dammam 34212, Saudi Arabia,; 3 PhD, Child Development and Exercise Center, Wilhelmina Children’s Hospital, University Medical Center Utrecht, Heidelberglaan 100, Utrecht, the Netherlands.

**Keywords:** caloric control, cardiovascular fitness, football exercise, musculoskeletal fitness, type 1 diabetes mellitus, adolescent

## Abstract

**Objective:**

The current study investigated the influence of recreational football exercise integrated with caloric control on the health-related fitness in adolescent males with type 1 diabetes mellitus (T1DM).

**Methods:**

We divided the participants into four distinct groups. The first group played football and followed a diet plan (FDG), while the second group played only football (FG), meanwhile, the third group had only a diet plan (DG), and the fourth group represented the control group (CG). Every group consisted of 10 male participants. The FDG and FG played twice weekly 90 minutes of football for 12 weeks. Body mass, body mass index (BMI), abdominal endurance, explosive strength, handgrip strength, trunk flexibility, and estimated VO2 max were measured before and after the 12 weeks. Changes in these variables were considered significant if p ≤ 0.050 and the value of effect size (ES) ≤ 42 % or ES ≥ 58 %.

**Results:**

Both the DG and FDG experienced a significant decrease in both body mass and BMI. Moreover, only the FDG experienced significant increases in all muscular fitness parameters and an increase in estimated VO2 max. The FG experienced significant increases in explosive strength and handgrip strength.

**Conclusion:**

An intervention of 12 weeks of integrated football with caloric control offers the highest improvements in musculoskeletal and cardiovascular fitness in adolescent boys with T1DM, thus improving their overall healthrelated fitness.

## 1 Introduction

Type 1 diabetes mellitus (T1DM) is a chronic disease of absolute insulin deficiency causing a disturbance of all the metabolites; fat, proteins, and carbohydrates manifesting as high blood glucose, caused by autoimmune destruction of pancreatic beta cells [1,2]. T1DM can lead to various medical disorders including coronary artery disease, retinopathy, nephropathy, and neuropathy [2]. As there is no available remedy for T1DM apart from pancreas transplantation [1], individual with T1DM are, recommended several ways to manage their disease. These recommendations include managing diabetes using insulin, diet monitoring, and physical activity [3]. Of interest is how to improve the health of persons with T1DM. A health parameter needing improvement is health-related fitness as it consists of health components of physical fitness that help individuals perform daily activities without unnecessary tiredness [4]. Individuals with T1DM have a lower health related fitness compared to healthy individuals [5,6]. Nevertheless, the overall health-related fitness of individuals with T1DM can be improved with physical activity alone in at least four weeks depending on the physical activity [7-9]. However, it is not known if a combination of physical activity and caloric control will have a greater effect on overall health-related fitness. Despite the profound health benefits of physical activity on the affected people with T1DM, persons with T1DM face challenges in obtaining these benefits. Firstly, aerobic exercise using either treadmills or cycle ergometers is one of the main forms of exercise to study improvements in the health-related fitness of people with T1DM. Such exercises are not considered attractive to individuals with non-infectious diseases, thus, making them less enthusiastic to perform such exercises [10]. Additionally, persons with T1DM do not spend much time doing physical exercise as healthy individuals [11,12] due to their concern of experiencing hypoglycemia during and after a physical activity [12-15]. Thus, it will be important to determine the benefits of any recreational physical activity on their health-related fitness. The study can be used to motivate individuals with T1DM to engage regularly in physical activity as it has been reported that they need evidence to help them overcome their fears [16]. Given what is unknown and the challenges, the current study focuses on determining the impacts of a 12-week recreational football integrated with a caloric control regimen on the health-related fitness of adolescent males having T1DM. The study hypothesis was that the 12-week intervention will improve the health-related fitness parameters of the enrolled adolescents. In the published literature, this is the first study to our knowledge to use recreational football combined with caloric control to improve the health-related fitness of male adolescents with T1DM. The manuscript will first provide the methodology, and more details will be provided in the Supplementary File. Next, the results will be first presented and then discussed. The limitations of the study along with future study recommendations and the practical implications will be provided in the discussion before providing a conclusion.

## 2 Methods

### 2.1 Participants

Forty male adolescents (age = 12-18 years) having T1DM for more than one year were enrolled in the study. The participants were equally distributed into four groups through simple randomization. The first group played football and followed a diet plan (FDG), while the second group played only football (FG). The third group followed only a diet plan (DG), and the fourth group represented the control group (CG).

### 2.2 Dietary program

A professional medical nutritionist had designed a dietary regimen ranging from 1500-2200 kcal to help the participants in FDG and DG to attain or maintain a healthy BMI.

The participants could eat their meal choices following the regimen. Also, they were requested not to consume more than their daily number of calories.

### 2.3 Football exercise program

The football program had small-team games (three to five participants in each team, depending on the participant number coming to each session) played on an indoor football pitch. There were two games weekly for a total of 12 weeks (21st January – 10th April 2019) and each game had two halves of 45 minutes each. Participants were given a 10-minute warm-up and five minutes of cool-down before and after each game, respectively. The participants were requested to stay within 80 % of their maximum heart rate to prevent out of control hyperglycemia during the high-intensity exercise [17,18] and to play fairly and safely. To maintain inter-group blindness of the different regimens, the FDG and FG played at different times.

### 2.4 Blood glucose monitorin

Consensus statement guidelines for blood glucose monitoring were followed [19]. A glucometer (AccuChek Performa, Roche) was used for blood glucose monitoring instead of a continuous glucose monitor to ensure that all the participants had a uniform method of blood glucose measurement. For each participant, the blood glucose was checked immediately before, during, and then after the games, and corrective measures were taken for participants with readings less than 120 mg/dL or more than 270 mg/dL of blood glucose. Measures were also taken to prevent the participants from having nocturnal hypoglycemia. Any participant with hypoglycemic readings and expected to experience hypoglycemic levels at night was requested to reduce his daily basal (long-acting) insulin dose [20-22] by 15 %.

### 2.5 Health-related fitness measurement

The health-related fitness assessment was done at King Fahd University of Petroleum and Minerals KFUPM for all the participants before and after the program. The measured health-related fitness parameters included body size using the body mass and BMI in kg.m-2 , the cardiorespiratory fitness measured using the FitKids Treadmill Test Protocol [23] muscular endurance using the 60 s curl-up test, flexibility using the sit-and-reach box in cm, handgrip strength using a hydraulic handgrip dynamometer (Baseline®, U.S.A, Model 12-0246) in kg, and explosive strength using the standing broad jump test in m. The equipment used for the assessment was checked before conducting the tests. The body height and body mass were measured for each participant in cm and kg, respectively using the mechanical column scale with an eye-level beam (Seca GmbH & Co. KG, Hamburg, Germany). The measures were taken while the participants were wearing light clothing and were without shoes. The curl-up, handgrip, and flexibility tests followed the fourth edition of the ACSM healthrelated fitness testing guidelines [24], except that a metronome was not used for the curl-up test. The participants were requested to do the maximum number of curl-ups they could perform. The handgrip strength test results were the average of the best scores obtained from both hands after the participants squeezed the dynamometer twice. For the standing broad jump test, the participants were asked to stand behind a marked line and jump forward with their feet and try to land upright. The distance jumped was measured with an inelastic measuring tape attached to the playground. The test was performed at least twice, and the best result was taken. The Fitkids Treadmill Test Protocol [23] was used to estimate the VO_2_ max. The treadmill used for the Fitkids Treadmill Test was Matrix Ultimate Deck T5x Treadmill. The VO_2_max was estimated using the following formula:

VO_2_ max = -0.748 + (0.117×TTE) + (0.032×body mass) + 0.263

where TTE is the time taken to exhaustion.

### 2.6 Physical activity assessment

Physical activity was assessed within the 12 weeks of intervention using the Google FitTM application. The number of daily steps was used as the scale of assessment of physical activity performed daily. The participants downloaded the application on their cell phones and carried their phones wherever they were, even while at the training site.

### 2.7 Statistical analysis

We used SPSS 16 and R statistical packages [25] to evaluate the statistical data. R was used in case SPSS 16 could not provide some statistical outputs. Parametric and non-parametric tests were used whenever necessary. Both p-values and the common-language effect size (CLES) were used to judge if the changes were clinically significant. Table 1 summarizes the criteria for assessing the clinical significance of the effect of the interventions.

**Table 1.** Criteria for assessing clinical significance of the effect of the Interventions

	CLES	Clinical significance
1	p > 0.050 and 42 % < CLES < 58 %	Not clinically significant
2	p ≤ 0.050 and 42 % < CLES < 58 %	Not clinically significant
3	p > 0.050 and CLES ≤ 42.0 % or CLES ≥ 58.0 %	Unclear^a^
4	If p ≤ 0.050 and CLES ≤ 42.0 % or CLES ≥58.0 %	Clinically significant

a The lack of clarity implies that a larger sample size might have improved the chances of detecting any effect induced by the interventions

## 3 Results

Data of the 40 participants were analyzed. The participants in both football groups had an attendance ranging from 21 to 24 sessions out of 24 sessions. The results of the caloric-control regimen, nocturnal hypoglycemic episodes, and physical activity records are in the Supplementary File. In summary, two out of the three participants who were beyond their healthy target BMI range successfully attained their target BMI (Table S3). Moreover, those who were within their healthy BMI at the start of the program were still in their healthy BMI range (Table S3). All participants except two did not exceed four nocturnal hypoglycemic episodes (Table S2). Two participants recorded seven hypoglycemic events (Table S2). Lastly, the football groups completed on average around six times the daily number of steps that the DG and the CG completed in the 12 weeks (Table S4), meaning they performed more physical activity than the diet and control groups. The comparison of the baseline data among the groups and the pairwise comparison of the baseline data are shown in both Table 2 and Table 3, respectively.

**Table 2.** Baseline data of the participants (Mean (SD))

Variable	CG (N=10)	DG (N=10)	FG (N=10)	FDG (N=10)	F/H; p value (among groups)	Unbiased ES/ CLES (%)
Age (years)	14.4 (2.0)	15.6 (1.8)	17.8 (0.42)	14.5 (1.4)	Not compared	
*Anthropometric measures*						
Body mass (kg)	61.1 (7.5)	53.2 (6.6)	58.2 (11.2)	55.6 (11.6)	2.05; 0.14	11.8/61
Height (cm)	161 (8.55)	157 (9.73)	156 (9.90)	166 (10.0)	Not compared	
BMI (kg.m^-2^)*	23.46 (1.96)	21.65 (3.17)	23.71 (3.34)	19.93 (2.40)	4.82; 1.1×10^-2^	32.7/69
*Musculoskeletal fitness*						
60 s Curl up (repetitions)	22 (7)	22 (4)	22 (9)	22 (9)	0.02; 1.0	0.0/50
Sit and reach (cm)	11 (4)	10 (3)	8 (5)	7 (5)	1.94; 0.16	10.7/61
Handgrip strength (kg)	20.4 (6.6)	23.3 (7.4)	19.5 (6.5)	24.6 (9.1)	0.93; 0.45	0.0/50
Standing long jump (cm)	126.8 (18.3)	115.0 (15.0)	123.4 (22.7)	142.6 (31.0)	2.27; 0.11	13.9/62
*Cardiovascular fitness*						
VO2 Max (mL.kg^-1^•min^-1^) ^NP^	40.71 (5.53)	41.21 (6.42)	44.37 (6.78)	41.78 (5.75)	2.26; 0.52	0.0/50
Resting heart rate (bpm)	89 (7)	82 (8)	95 (12)	89 (7)	2.55; 8.5×10-2	16.5/63

* Significant difference (p ≤ 0.050, CLES ≤ 42.0 % and CLES ≥ 58.0 %); NP = Non-parametric test

**Table 3.** Pairwise comparison of baseline values (N= 10 per group)

	p-value (95% CI/Z); column vs row				CLES		
Variable	Group	DG	FG	FDG	DG	FG	FDG
*Anthropometric measures*							
Body mass (kg)	CG	9.3×10^-2^ (-1, 17)	0.90 (-9.3, 15)	0.59 (-7, 18)	80.5	56.5	65.5
	DG		0.63 (-17, 6.9)	0.94 (-15, 9.9)		35.0	42.0
	FG			0.95 (-12, 17)			56.5
BMI (kg.m-2)	CG	0.44 (-1.6, 5.2)	1.0 (-3.8, 3.3)	1.0×10^-2^ (0.75, 6.3)	69.0	47.5	88.0
	DG		0.51 (-6.2, 2.1)	0.53 (-1.9, 5.3)		33.5	67.0
	FG			4.5×10^-2^ (0.075, 7.5)			82.0
*Musculoskeletal fitness*							
60 s Curl up (repetitions)	CG	1.0 (-8, 7)	1.0 (-10, 9)	1.0 (-11, 9)	50.5	50.5	42.5
	DG		1.0 (-9, 8)	1.0 (-10, 8)		50.5	38.0
	FG			1.0 (-11, 11)			44.0
Sit and reach (cm)	CG	0.88 (-3, 6)	0.51 (-3, 8)	0.14 (-1, 10)	62.0	68.0	75.0
	DG		0.78 (-3, 6)	0.25 (-2, 8)		60.0	71.0
	FG			0.82 (-4, 8)			60.0
Handgrip strength (kg)	CG	0.79 (-12, 6)	0.99 (-7.4, 9.2)	0.65 (-14, 5.9)	40.5	54.5	36.5
	DG		0.63 (-5.1, 13)	0.99 (-12, 9.2)		63.5	47.0
	FG			0.49 (-15, 5)			34.0
Standing long jump (cm)	CG	0.42 (-9.5, 33)	0.98 (-23, 30)	0.53 (-49, 17)	69.0	54.5	33.5
	DG		0.77 (-33, 16)	0.10 (-60, 4.3)		40.0	23.5
	FG			0.42 (-54, 15)			29.0
*Cardiovascular fitness*							
VO2 Max (mL.kg^-1^•min^-1^) ^NP^	CG	1.0 (-5.74×10^-2^	1.0 (-1.32)	1.0 (-0.31)	50.0	31.0	47.0
	DG	1.0 (-1.26)	1.0 (-0.25)			35.0	46.0
	FG		1.0 (1.01)				63.0
Resting heart rate (bpm)	CG	0.20 (-2.6, 17)	0.63 (-18, 7.3)	1.0 (-8.2, 8.8)	77.0	37.5	50.5
	DG		7.3×10-2 (-26, 0.93)	0.24 (-17, 3)		19.0	23.0
	FG			0.60 (-7.1, 18)			63.5

† Significant difference (p ≤ 0.050, CLES ≤ 42.0 % and CLES ≥ 58.0 %); NP = Non-parametric statistical test used; CLES = Common language effect size

The baseline differences were considered significant as the CLES of the pairwise comparisons were nonnegligible despite the differences being statistically insignificant. Table 4 shows the results of the compared baseline and post-intervention data and the results of the compared net changes among the groups. The group comparisons of the overall changes are shown in Table 5. The pairwise comparisons ensured that the changes also consider the CG.

**Table 4.** Baseline vs post comparisons and comparison of mean difference results among the groups (N= 10 per group)

Variable	Group	Pre (Mean (SD))	Post (Mean (SD))	Mean Difference (SD)	p value (95 % CI) or p value (Z on negative ranks) ^a^	CLES^a^	df from Welch ANOVA (among, within) ^b^	F-statistic or H-statistic; p value (among groups) ^b^	Unbiased ES/CLES (%) ^b^
Age (years)	CG	14.4 (2.0)	14.8 (1.9)	Not compared					
	DG	15.6 (1.8)	16.1 (1.7)						
	FG	17.8 (0.42)							
	FDG	14.5 (1.4)	14.8 (1.3)						
*Anthropometric measures*									
BMI (kg.m^-2^	CG	23.46(1.96)	24.11	0.65 (1.07)	5.9×10^-2^ (-1.886)	60.5	(3, 19.507)	18.29; 6.9×10^-06^*	68.8/81
	DG†	21.65(3.17)	-2.16 (0.98)	7.7×10^-5^ (-2.84, -1.43)	29.0				
	FG	23.71 (3.34)	24.00 (2.34)	0.29 (0.59)	3.7×10^-2^ (0.03, 0.68)	54.5			
	FDG	19.93(2.40)	19.15(2.34)	-0.78 (0.66)	1.7×10^-2^ (-1.16, -0.15)	42.0			
*Musculoskeletal fitness*									
	CG†	22 (7)	19 (7)	-3 (3)	1.8×10^-2^ (-2.360)	40.5	(3, 19.903)	15.97; 1.6×10^-05^*	65.3/80
	DG	22 (4)	21 (3)	-1 (3)	0.24 (-3, 1)	45.0			
	FG	22 (9)	23 (7)	1 (3)	0.44 (-0.776)	54.0			
	FDG†	22 (9)	29 (10)	7 (3)	9.4×10^-5^ (5, 9)	77.5			
	CG	11 (4)	10 (3)	-1 (2)	8.9×10-2 (-1.699)	42.0	Kruskal- Wallis	29.92; 1.4×10^-6^*	74.8/83
	DG	10 (3)	10 (3)	0 (1)	0.56 (-0.9, 0.5)	48.5			
	FG†	8 (5)	10 (3)	2 (2)	4.0×10^-3^ (0.8, 3.2)	62.5			
	FDG†	7 (5)	20 (6)	13 (4)	6.3×10^-6^ (10.0, 16.4)	94.5			
	CG	20.4 (6.6)	18.6 (5.9)	-1.8 (2.6)	5.4×10^-2^ (-3.6, 0.0)	42.0	Kruskal- Wallis	20.13; 1.6×10^-4^*	47.6/74
	DG	23.3 (7.4)	22.5 (7.0)	-0.8 (2.3)	0.30 (-2.4, 0.8)	47.0			
	FG†	19.5 (6.5)	22.5 (5.9)	3.0 (2.2)	2.0×10^-3^ (1.4, 4.6)	62.5			
	FDG†	24.6 (9.1)	31.4 (6.0)	6.8 (7.1)	1.4×10^-2^ (1.7, 11.9)	73.5			
	CG†	126.8 (18.3)	121.4 (19.8)	-5.4 (5.9)	1.8×10^-2^ (-9.6, -1.2)	37.0	Kruskal- Wallis	27.68; 4.2×10^-6^*	
	DG	115.0 (15.0)	113.4 (15.5)	-1.6 (4.7)	0.31 (-5.0, 1.8)	45.5			
	FG†	123.4 (22.7)	129.8 (17.4)	6.4 (7.6)	2.6×10^-2^ (1.0, 11.8)	61.5			
	FDG†	142.6 (31.0)	162.6 (32.3)	20.0 (12.1)	5.5×10^-4^ (11.3, 28.7)	67.5			
*Cardiorespiratory fitness*									
VO^2^ Max (mL.kg^-1^. min^-1^)	CG	40.71 (5.53)	39.48 (4.29)	-1.23 (1.44)	2.4×10^-2^ (-2.27, -0.20)	44.0	Kruskal- Wallis	20.13; 1.6×10^-4^*	47.6/74
	DG	41.21 (6.42)	41.67 (6.25)	0.45 (0.78)	9.8×10^-2^ (-0.10, 1.01)	55.0			
	FG	44.37 (6.78)	44.31 (7.06)	-0.06 (2.06)	7.4×10^-2^ (-1.784)	57.0			
	FDG†	41.78 (5.75)	41.78 (5.75)	5.38 (5.46)	1.2×10^-2^ (1.47, 9.29)	76.0			
Resting Heart Rate (bpm)	CG	89 (7)	93 (6)	3 (7)	0.19 (-2, 8)	64.0	(3, 16.999)	32.58; 2.9×10^-07^*	81.9/86
	DG†	82 (8)	89 (10)	7 (6)	5.0×10^-3^ (3, 11)	76.0			
	FG†	95 (12)	85 (7)	-9 (6)	1.0×10- (-13, -5)	26.5			
	FDG†	89 (7)	79 (6)	-10 (2)	2.6×10^-8^ (-11, -9)	13.5			

a Result from post vs baseline comparison. ^b^Result from comparing mean difference of each group.

* Statistically significant difference (p < 0.05)

† Significant difference (p ≤ 0.050, CLES ≤ 42.0 % and CLES ≥ 58.0 %)

ES = Effect size; CLES = Common language effect size

**Table 5.** Pairwise comparison of the mean difference results (N= 10 per group)

	p-value (95% CI/Z); column vs row				CLES		
Variable	Group	DG	FG	FDG	DG	FG	FDG
*Anthropometric measures*							
Body mass (kg)	CG	2.2×10^-5^ (3.9, 9.7)†	0.748 (-1.7, 3.5)	1.6×10^-2^ (0.6, 6.5)†	99.0	63.5	85.5
	DG		2.0×10^-6^ (-7.9, -3.9)†	7.5×10^-3^ (-5.7, -0.79)†		0.0	9.5
	FG			1.3×10^-2^ (0.52, 4.8)†			89.0
BMI (kg.m^-2^)	CG	5.2×10^-5^ (1.5, 4.1)†	0.79 (-0.77, 1.5)	1.3×10^-2^ (0.27, 2.6)†	98.0	63.0	92.0
	DG		3.6×10^-5^ (-3.5, -1.4)†	9.5×10^-3^ (-2.5, -0.31)†		1.0	9.0
	FG			6.6×10^-3^ (0.27, 1.9)†			85.0
*Musculoskeletal fitness*							
60 s Curl up (repetitions)	CG	0.64 (-5, 2)	0.1 (-7, 1)	1.8×10^-5^ (-13, -5)†	33.0	22.0	0.0
	DG		0.46 (-6, 2)	7.6×10^-5^ (-12, -4)†		26.5	0.0
	FG			2.8×10^-3^ (-10, -2)†			4.5
Sit and reach (cm) ^NP^	CG	1.0 (-0.510)	5.4×10^-2^ (-2.610)	6.0×10^-6^ (-4.893)†	38.0	8.0	0.0
	DG		0.21 (-2.100)	7.1×10^-5^ (-4.382)†		10.5	0.0
	FG			0.13 (-2.283)			0.0
Handgrip strength (kg) ^NP^	CG	1.0 (-0.509)	3.2×10^-2^ (-2.783)†	7.2×10^-4^ (-3.848)†	38.0	8.0	11.0
	DG		0.14 (-2.274)	5.0×10^-3^ (-3.339)†		10.5	11.0
	FG			1.0 (-1.065)			20.5
Standing long jump (cm) ^NP^	CG	1.0 (-0.891)	3.3×10^-2^ (-2.780)†	7.8×10^-6^ (-4.840)†	29.5	9.5	0.0
	DG		0.35 (-1.888)	4.7×10^-4^ (-3.949)†		15.0	0.0
	FG			0.24 (-2.061)			8.5
*Cardiovascular fitness*							
VO2 Max (mL.kg^-1^•min^-1^) ^NP^	CG	0.17 (-2.200)	0.29 (-1.970)	4.6×10^-5^ (-4.476)†	12.0	15.0	10.0
	DG		1.0 (0.230)	0.14 (-2.276)		54.0	10.0
	FG			7.3×10^-2^ (-2.507)			9.0
Resting heart rate (bpm)	CG	0.56 (-12, 4.3)	2.4×10^-3^ (4.2, 20)†	6.1×10^-4^ (6.5, 20)†	35.0	94.5	100
	DG		5.5×10^-5^ (8.6, 24)†	1.7×10^-5^ (11, 23)†		98.5	100
	FG			0.96 (-5, 7)			70.5

† Significant difference (p ≤ 0.050, CLES ≤ 42.0 % and CLES ≥ 58.0 %)

NP = Non-parametric statistical test used; CLES = Common language effect size

### 3.1 Anthropometric measures

We found a significant decrease in body mass in the DG and FDG after 12 weeks (Table 4) and they were significant when considering the CG (Table 5). The BMI significantly decreased in 12 weeks in the DG (Table 4) after considering the CG (Table 5). The decrease in BMI in the FDG after 12 weeks was not significant (Table 4), even though the decrease was significantly different from the rise in BMI in the CG (Table 5). The change in body size in the DG was significantly different from the changes in body size in the FDG and FG (Table 5). Moreover, we did not observe significant changes in participant body mass in the FG. To summarize, the magnitude of reduction in body size after the pairwise comparisons was as follows: DG > FDG > FG > CG.

### 3.2 Musculoskeletal fitness

All the musculoskeletal fitness parameters of the FDG significantly increased after 12 weeks (Table 4) even after considering the CG (Table 5). The FG also showed significant changes in handgrip strength and explosive power (Table 4) even after considering the CG (Table 5). The flexibility of the FG did not change (Table 4), while the increase in abdominal endurance in the FG (Table 4) was not clear after considering the CG (Table 5). The FDG significantly surpassed the DG in all musculoskeletal fitness parameters (Table 5). Though the FDG also surpassed the FG in all parameters (Table 5), the differences between both groups were not clear except with abdominal endurance (Table 5). Meanwhile, there was no significant change in musculoskeletal fitness in the DG, and there was a significant decrease in the flexibility and explosive strength in the CG (Table 4).

### 3.3 Cardiovascular fitness

There was a significant increase in the estimated VO2 max in the FDG after 12 weeks (Table 4) even after considering the CG (Table 5). The DG and FG did not show a significant change in the estimated VO2 max (Table 4).

## 4 Discussion

The study aimed to evaluate the effects of a 12-week recreational football integrated with a caloric control regimen on the health-related fitness of adolescent males with T1DM. Each health-related fitness component shall be discussed separately. Anthropometric measures the interventions affected the body size of the caloric control groups, with the caloric control only group having a greater change than the football-with-caloric control group. However, the caloric control-only group did not necessarily experience a better improvement in body composition than the football-with-caloric control group as there were most likely changes in the musculoskeletal mass of the participants due to the football games [26]. Similar changes in body size were reported in people having type 2 diabetes mellitus (T2DM) who went through a football with diet intervention and a diet-only intervention, the authors showed that their football with diet group obtained greater increases in lean body mass compared to their dietonly group [27]. Thus, it is inferred that the caloric control-only group in the current study experienced the greatest reduction in body mass because they did not do enough physical activity to significantly increase their lean mass. An increase in musculoskeletal mass is important for persons with T1DM as it will prevent the development of insulin resistance, especially in those who are obese, moreover, people with T1DM will have a reduced risk of suffering from cardiovascular diseases and will have an increase in skeletal muscle mass [28].

Even though there was no significant reduction in the body size of the football-only group, it cannot be concluded that there was no significant change in their body composition. The lean mass of the football-only group may have increased due to the football program. Unfortunately, we did not measure lean body mass in this study to confirm the inference. To summarize, caloric control reduced the body mass of the participants. Moreover, football most likely increased the lean mass of the participants who controlled their caloric intake.

### 4.1 Musculoskeletal fitness

The football with caloric control group experienced significant increases in all the musculoskeletal fitness parameters after 12 weeks, while the football-only group had increases in explosive strength and handgrip strength. Moreover, the football with caloric control group had higher gains than the football-only group in abdominal endurance. Thus, both football groups had improvements in musculoskeletal fitness, in agreement with previous studies (Absil H et al., 2019), though the football with caloric control group had better gains. The improvements in explosive strength in both groups are attributed to the growth of muscle fiber size induced by periods of acceleration and deceleration during the football game [29]. Football also induces growth in abdominal muscles [30], thus explaining the improvements in abdominal muscle endurance in the football-with-caloric control group. The football with caloric control group having better gains than the football-only group in musculoskeletal fitness is similar to studies that used resistance training with and without caloric control in non-obese young adults [31] and obese old men [32]. As both football and resistance training have similar effects on musculoskeletal fitness, the conclusions from the resistance training studies will help explain the current results. The resistance training studies show that high adiposity reduces the gains in muscular strength despite a similar increase in muscle size. Thus, the football with caloric control group obtained higher gains in musculoskeletal fitness probably due to a reduction in adiposity. The reduction in adiposity was due to caloric control. The inference is supported by the decrease in body mass of the football-with-caloric control group and an insignificant change in body mass of the football-only group. The caloric control-only group did not experience significant changes, implying that caloric control was not enough to improve the muscular fitness and flexibility of the participants. The control group experienced a decrease in flexibility and explosive strength, meaning they experienced a decrease in musculoskeletal fitness.

### 4.2 Cardiovascular fitness

Only the football with caloric control group experienced an increase in estimated VO_2_max. The absence of a significant change in the VO_2_ max of the football-only group seems to indicate that recreational football alone is insufficient to improve the aerobic fitness of male adolescents with T1DM in 12 weeks. The conclusion disagrees with studies that showed improvements in cardiorespiratory fitness after at least 12 weeks of moderate to high-intensity exercise [33], or after four weeks of high-intensity interval training (HIIT) [10]. The disagreement may arise from the fact that the current study had adolescents while the cited studies included adults with diabetes. As the only difference between the groups was the presence of caloric control, it could mean that adolescent boys with T1DM require caloric control to experience greater improvements in cardiorespiratory fitness. The DG did not experience any change in cardiorespiratory fitness. People with T2DM did not also experience improvements in cardiorespiratory fitness when solely on caloric restriction, but when it was supplemented with football [30].

To summarize, a mixture of caloric control and football helped the adolescent boys with T1DM to greatly improve their cardiovascular fitness in 12 weeks.

### 4.3 Limitations

The study had the following limitations: (1) Small sample size, making it difficult to detect significant changes. (2) Only adolescent boys were included in the study, thus reducing the generalizability of the study. (3) Incomplete and inaccurate dietary records, though it was expected [34] and controlled. (4) Not using a continuous heart rate monitor to measure the heart rates of the participants during the game. (5) Baseline physical activity was not documented. It is recommended to perform the study with a larger sample size that includes people of different sexes and age groups.

### 4.4 Practical implications

Though physical activity improves the health-related fitness of individuals with T1DM, the current study shows that combining both a recreational activity with caloric control will lead to greater improvements in fitness in male adolescents with T1DM than physical activity alone. Moreover, male adolescents with T1DM may get such benefits without fearing hypoglycemia if they take the necessary precautions to ensure their blood sugar levels are at appropriate levels before they engage in physical activity. The assessment is based on the fact that only two participants exceeded four nocturnal hypoglycemic episodes during the 12 weeks. Lastly, the study adds evidence to the recommendation that football provides health benefits for individuals having non-infectious diseases [12,35].

### 4.5 Conclusion

This study demonstrated that an intervention consisting of combined football with caloric control offers the best improvements in health-related fitness for adolescent boys with T1DM.

## Abbreviations

T1DM: Type 1 Diabetes Mellitus

FDG: Football and Diet Group

DG: Diet Group

FG: Football Group

CG: Control Group

BMI Body: Mass Index

VO2 max: Maximum Volume of O2 USED DURING EXERCISE
